# Job satisfaction among rehabilitation professionals employed in public health facilities in KwaZulu-Natal

**DOI:** 10.4314/ahs.v23i2.87

**Published:** 2023-06

**Authors:** N Makholwa, B Tlou, TP Dlungwane

**Affiliations:** College of Health Sciences, School of Nursing and Public Health, Discipline of Public Health Medicine, University of KwaZulu-Natal, South Africa

**Keywords:** Job satisfaction, rehabilitation professionals, public health facilities

## Abstract

**Background:**

Job satisfaction is essential in stimulating productivity and efficiency in the health sector. Dissatisfied employees are likely to leave, which places an added burden considering the shortage of health workers. Rehabilitation professionals form a critical component of the public health workforce.

**Objective:**

The aim of the study was to document the level of job satisfaction and factors associated with job satisfaction among rehabilitation professionals employed in public health facilities in KwaZulu-Natal.

**Methods:**

A cross-sectional survey was conducted. A self-administered questionnaire was utilized. A Chi-square test and logistic regression were used to assess associations and to identify factors associated with job satisfaction. A p-value less than 0.05 was deemed statistically significant.

**Results:**

Most participants (59%) reported a low level of overall job satisfaction. The participants were dissatisfied about not getting recognition for work related to their specific professions (61.3 %) and not being considered for career advancement (74.3 %). In addition, inadequate financial rewards (87.2%) and benefits (71.3%) were also linked to low job satisfaction.

**Conclusion:**

Participants displayed a low level of job satisfaction. Rehabilitation services should be prioritized, and appropriate recognition should be granted to rehabilitation professionals in order to enhance job satisfaction.

## Introduction

Job satisfaction is perceived as the employee's level of contentment and fulfilment derived from one's job^1^. Job satisfaction is an essential determinant of health professionals' performance and turnover globally 2,3. Healthcare workers' level of satisfaction is vital to improve staff retention and quality of health care delivery 2. The motivation of healthcare workers can lead to high patient satisfaction and increase the productivity and efficiency of health care systems^4^,^5^.

Job satisfaction is influenced by a safe working environment, payment and compensation, career development and training, supportive leadership, good interpersonal relationships, and teamwork^2^,^6^. Conversely, job dissatisfaction is associated with workload, lack of training opportunities, low salaries, and financial rewards. Job dissatisfaction could result in employee absenteeism and turnover, a decline in productivity, and poor service delivery.

Low job satisfaction amongst public health workers in low-and middle-income countries has been reported^3^,^7^,^8^. In low- and middle-income countries, employees' job satisfaction is critical since low staff retention and a significant shortage of healthcare workers^2^. A study comparing job satisfaction and intention to leave among health workers in Tanzania, Malawi, and South Africa (SA) found that SA had the lowest job satisfaction and highest intention to leave 3. Health professionals working in public hospitals were less satisfied than those working in clinics and health centers 3

Rehabilitation professionals play a critical role within a health care system, both in the public and private sector 9. Rehabilitation professionals include physiotherapists, occupational therapists, speech therapists. They are the key role players in preventing and managing the disease that can lead to disability and improve patients' quality of life by restoring their independence through rehabilitation. Studies that have been conducted in SA have focused on one cadre of rehabilitation professionals, which is occupational therapists 10,11. There is a lack of literature regarding job satisfaction among the other rehabilitation professional cadres in SA. The aim of the study was to document the level of job satisfaction and factors associated with job satisfaction among rehabilitation professionals employed in public health facilities in KwaZulu-Natal.

## Methods

A cross-sectional study design was implemented. Rehabilitation professionals who were permanently working in public hospitals between January 2018 and December 2018 in KZN were invited to participate. The study population consisted of physiotherapists, occupational therapists, audiologists, and speech therapists. A standardized self-administered questionnaire was used to determine the factors influencing job satisfaction among rehabilitation professionals working in public hospitals. The questionnaire was previously used in a study to measure job satisfaction among healthcare professionals in sub-Saharan Africa. A set of 29 items measured on a 4-point Likert scale was used to measure job satisfaction (ranging from 1=strongly agree to 4=strongly disagree). Participants were deemed satisfied with their job if their responses showed satisfaction in 80% of the questions asked. The researcher distributed questionnaires, and an electronic questionnaire was sent for those that were difficult to reach. A pilot study was done with ten rehabilitation professionals from another province to ensure that it was user-friendly.

Data was captured onto an Excel spreadsheet and then imported to Statistical Package of Social Sciences (SPSS) version 27. A p-value of less than 0.05 was deemed as statistically significant. Categorical variables were presented using proportions and frequency distribution tables, while numerical variables were summarized using measures of central tendency(means) and dispersion (standard deviations). Multiple logistic regression was used to identify factors associated with job satisfaction.

Ethics approval was obtained from UKZN Biomedical Research Ethics Committee BE 569/17) and from the provincial KZN Department of Health (HRKM Ref: 462/17). Participants who agreed to take part in the study signed informed consent. Study participants' information was kept confidential by not capturing codes as identifiers on questionnaires.

## Results

A total of 181 study participants were reached at the time of data collection, 109 questionnaires were adequately completed yielding a response rate of 60.2 %. The mean age for the study participants was 27 years (SD = 27.10). Majority of the participants were females (n =91; 83.5 %) and were aged between 20 to 30 (n=69; 3.3%). More than half of the participants were physiotherapists (n=63; 57.8 %), at entry-level (n=77; 70.6%) and worked in rural areas (n=66; 56.1%). A high proportion of participants had work experience of between one and five years (n=49; 44 %) and worked in the district level of care (n=48; 44 %). The demographic profile of the participants is presented in Table 1 below.

**Table 1:** Demographic profile of participants (n=109)

Demographic characteristics	Frequency	Percentage (%)
**Age**		
20-30	69	63.3
31-40	11	10
41-50	12	11
>50	17	15

**Gender**		
Male	18	16
Female	91	83.5

**Marital status**		
Single	61	56
Married	48	44

**Profession**		
Physiotherapist	63	57.8
Occupational Therapist	19	17.4
Audiologist	18	16.5
Speech therapy	9	8.3

**Work experience (years)**		
1-5	49	45
6-15	48	44
>15	12	11

**Job level**		
Assistant director	11	10.1
Chief	21	19.3
Entry-level	77	70.6

**Place of work**		
Rural	60	55
Urban	47	43.1
Missing	2	1.9
**Level of care**		
Tertiary	16	14.7
Regional	45	41.3
District	48	44

**Figure 1:**
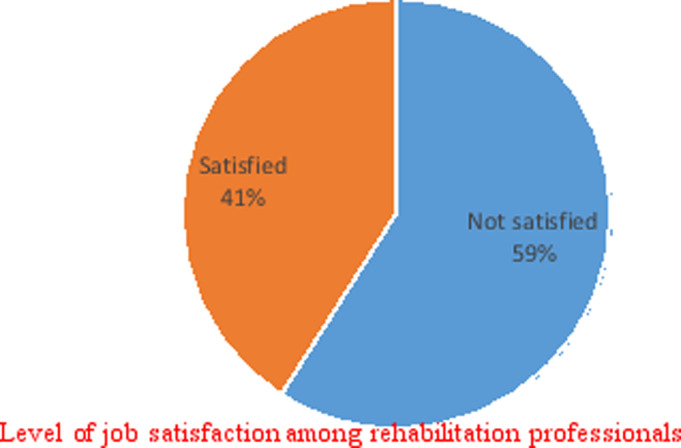
Level of job satisfaction

The majority (59%) of the study participants reported dissatisfaction with their job, while 41% were satisfied (Fig 1).

The majority of the participants were dissatisfied about not getting recognition for work related to their specific professions (61.3 %), not receiving continuity of education (54.6 %), and not being considered for career advancement (74.3 %). The majority of participants reported dissatisfaction with their lack of recognition from the provincial and national levels (76.9 %). In addition, they were not satisfied with the salary (87.2%) and benefits (71.3%). The majority of participants were satisfied with their job description since it was aligned with their responsibilities (91 %), their job was making a difference in the lives of others (91.6 %), and they were satisfied by the encouragement they received to carry out their duties (65.7 %).

Age and level of care were significantly associated with job satisfaction amongst rehabilitation professionals. Rehabilitation professionals within the 20 - 30(aOR = 0.056 [95% CI: 0.003, 1.019]) and 31 - 40(aOR = 0.169 [95% CI: 0.009, 3.045]) age groups were less satisfied when compared to those older than 50 years. Contrarily, rehabilitation professionals within the 41 - 50 age group were approximately two times (aOR = 1.727 [95% CI: 0.127, 23.444]) more likely to be satisfied when compared to those older than 50 years. In addition to that, rehabilitation professionals based at the regional (aOR = 0.086 [95% CI: 0.012, 0.630]) and district (aOR = 0.079 [95% CI: 0.012, 0.511]) health facilities were less satisfied as compared to those based at tertiary health facilities. On the other hand, marital status was not significantly associated with job satisfaction. Still, single rehabilitation professionals were three times (aOR = 3.032 [95% CI: 0.683, 13.466]) more likely to be satisfied when compared to those who are married. Similarly, women were approximately 1.5 times (aOR = 1.510 [95% CI: 0.40, 5.692]) more likely to be satisfied when compared to men. In addition, rehabilitation professionals based in rural areas were less satisfied (aOR = 0.778 [95% CI: 0.276, 2.189]) when compared to those found in urban areas. Lastly, rehabilitation professionals with more than 15 years of working experience were approximately 1.6 times (aOR = 1.555 [95% CI: 0.043, 56.249]) more likely to be satisfied when compared to those with less working experience of fewer than five years. Table 3 below summarises the multiple logistic regression results.

**Table 3:** Association between sociodemographic and rehabilitation professional's job satisfaction

Variable	Categories	Odds Ratio	95 % CI	*p-value*
**Age**	20 - 30	0.056	0.003 – 1.019	*0.050*
	31 - 40	0.169	0.009 – 3.045	*0.228*
	41 - 50	1.727	0.127 – 23.444	*0.681*
	>50	1		
**Gender**	Female	1.510	0.400 – 5.692	*0.543*
	Male	1		
**Marital Status**	Single	3.032	0.683 – 13.466	*0.145*
	Married	1		
**Profession**	Physiotherapist	0.423	0.057 - 3.158	*0.402*
	Occupational Therapist	0.754	0.089 – 6.382	*0.796*
	Audiologist	1.474	0.174 – 12.449	*0.796*
	Speech Therapist	1		
**Place of work**	Rural	0.778	0.276 – 2.189	*0.634*
	Urban	1		
**Job level**	Chief	2.653	0.122 – 57.652	*0.535*
	Production level	7.806	0.449 – 135.626	*0.158*
	Director	1		
**Level of care**	Regional	0.086	0.012 – 0.630	*0.016*
	District	0.079	0.012 – 0.511	*0.008*
	Tertiary	1		
**Work Experience(years)**	1- 5	1		
	6 - 15	0.757	0.195 – 2.934	*0.687*
	>15	1.555	0.043 – 56.249	*0.810*

## Discussion

The current study aimed to establish job satisfaction and factors influencing job satisfaction among rehabilitation professionals working for KZN public health institutions. The findings of this study indicated that the majority of rehabilitation professionals were dissatisfied with their jobs. More than half (59%) of the rehabilitation professionals were dissatisfied with their jobs, and the remaining 41% were satisfied with their jobs. A study conducted in Free State province, SA, reported that 55.88% of occupational therapists were dissatisfied with their current jobs10. In addition, a low level of satisfaction among healthcare workers has been reported in other studies 3,12,13.

South Africa has not made adequate investments in rehabilitation services^14^. Rehabilitation professionals feel that the profession is undervalued and not granted the status it deserves^10^. Rehabilitation professionals are part of a multidisciplinary team within the health sector that comprises physiotherapists, occupational therapists, speech therapists, and audiologists. They play a significant role in alleviating disability and improving life expectancy by preventing unnecessary complications resulting from illness or injury, which may eventually lead to premature disability death 3,15. Participants in this study reported that provincial and national levels' lack of recognition of their professions influenced dissatisfaction with their current jobs.

Career progression and development are essential in maintaining high satisfaction levels amongst health workers^4^. Workers who are provided with training and development opportunities demonstrate high levels of job satisfaction^16^. However, inadequate career paths and lack of continued education led to dissatisfaction^17^. Participants in this study identified career advancement and continuing education as important factors for job satisfaction amongst rehabilitation professionals in KZN, SA. Organizations that prioritize employee career management and development are likely to increase job satisfaction.

Compensation is in the form of financial allowances and rewards that influence the worker's perception of the job and, therefore, can determine an employee's level of job satisfaction^18^. Pay practices are crucial for organizations to retain high-quality employees^19^. The participants in this study reported there were not satisfied with their current salary and benefits. These findings are comparable with some studies conducted amongst health workers in SA^1^,^20^. The results of the studies amongst health workers from different provinces within SA highlighted that salary levels and structure was not enough to meet their financial needs and day-to-day living expenses^1^,^11^,^20^. Health care organizations need to devise innovative reward systems to attract and retain a high calibre of health care workers^5^,^15^.

The majority of the participants in this study were satisfied with the work environment, workload, and job security. Having a good working environment and job security is essential to enhance job satisfaction amongst health workers. Satisfied workers tend to be more productive and committed to the employer^2^. Evidence suggests that when health care workers are satisfied in their jobs, the patients receive good service in return^2^,^6^. In addition, the health sector saves money that could be spent on litigation and medico-legal expenses^2^,^6^.

Age and level of care were significantly associated with job satisfaction amongst rehabilitation professionals. Rehabilitation professionals within the 20 - 30(aOR = 0.056 [95% CI: 0.003, 1.019]) and 31 - 40(aOR = 0.169 [95% CI: 0.009, 3.045]) age groups were less satisfied when compared to those older than 50 years. These results are also in line with studies done by Munyewende et al. (2014) and Blaauw et al. (2013). They reported that dissatisfaction among younger professionals was associated with their intention to leave. Job dissatisfaction among young professionals is a challenge since they contribute more to the workforce than older professionals who will retire soon 5.

## Limitations

This is a cross sectional study therefore it did not give information over a period of time. Since it was a self-administered questionnaire, it is therefore possible that the participants might have over- or under-reported their level of satisfaction. The findings of the study may not be generalised to rehabilitation professionals working in the private sector.

## Recommendations

Rehabilitation services should be prioritized, and appropriate recognition should be granted to these cadres. Furthermore, the issues relating to compensation, career development, and progression should be given priority by health management within KZN. A further study should be conducted incorporating rehabilitation professionals from both the public and private sectors. In addition, qualitative research should explore how the factors influence productivity and staff retention in the health sector.

## Conclusion

The participants displayed a low level of job satisfaction. Lack of recognition for rehabilitation professionals, career advancement and progression, salary levels, and structure were the leading causes for job satisfaction amongst the study participants. In addition, age and level of care were significantly associated with job satisfaction amongst rehabilitation professionals.

## Figures and Tables

**Table 2:** Rehabilitation professional's job satisfaction characteristics. Interpersonal / Intrinsic factors

Characteristic(item)	Satisfied (%)	Dissatisfied (%)
**Interpersonal / Intrinsic factors**		
1. Do you get recognition for work related to your profession?	38.7	61.3
2. Do you get support and assistance from your colleagues?	90.7	9.3
3. Do you receive continuity of education?	45.4	54.6
4. Are you considered for advancement in your career?	25.7	74.3
5. Are you satisfied with what you have accomplished since you started working in the public sector?	57.5	42.5
6 Do you receive encouragement from your patients?	89.9	10.1

Environmental / extrinsic factor		
Characteristic(item)	Satisfied (%)	Dissatisfied (%)
**Workload** 1. I am satisfied with my working schedule.	78.9	21.1
2. I can balance between seeing patients and other activities.	70.4	29.6
3. I see enough and an acceptable number of patients per day.	66.7	33.3
**Work environment**		
1. I am provided with the necessary equipment to do my duties.	50.9	49.1
2. I have an opportunity to utilize my skills and abilities in my working setup.	72	28
3. My Department is safe, spacious, and accessible to my patients.	65.4	34.6
**Management**		
1. Do you communicate frequently and enough with your immediate Manager?	70.4	29.6
2. Do you get assistance in work-related issues?	69.4	30.6
3. Are you involved in decision making in your department?	69.4	30.6
**Organisational level**		
1. Did you know and understand the vision, mission, and core values of your institution?	88.9	11.1
2. Are you clearly informed of the Department of Health policies?	73.1	26.9
3. Is your job included in national, provincial, and institutional goals?	58.7	41.3
4. Is your job well aligned with national and provincial health indicators?	57.5	42.5
5. Do you think your job is well recognized at the provincial and national levels?	23.1	76.9
**Task**		
1. Does your job description clearly indicate your responsibilities and ability?	91.6	8.4
2. Do you think your job makes any difference in the lives of others?	99.1	0.9
3. Do you think you are well equipped to use any appropriate equipment needed for your job?	85.3	14.7
4. Do you feel encouraged to carry on with your daily duties?	65.7	34.3
**Salary and benefits**		
1. Do you think the salary you earn is compensation enough for the work you do?	12.8	87.2
2. Is your salary level able to meet your financial needs?	11.9	88.1
3. Are you satisfied with the benefits that come with your job?	28.7	71.3
**Job security**		
1. Do you have concerns about losing your job?	74.3	26.7
2. Is your salary paid on time?	98.1	1.9
3. Do you feel you have stability in your job?	82.6	17.4
